# Draft Genome Sequences of Four *Alteromonas macleodii* Strains Isolated from Copper Coupons and Grown Long-Term at Elevated Copper Levels

**DOI:** 10.1128/genomeA.01311-16

**Published:** 2016-11-23

**Authors:** Kathleen D. Cusick, Jason R. Dale, Brenda J. Little, Justin C. Biffinger

**Affiliations:** a National Research Council, Washington, District of Columbia, USA; b U.S. Naval Research Laboratory, Washington, District of Columbia, USA; c U.S. Naval Research Laboratory, Stennis Space Center, Mississippi, USA

## Abstract

*Alteromonas macleodii* is a marine bacterium involved in the early stages of biofouling on ship hulls treated with copper as an antifouling agent. We report here the draft genome sequences of an *A. macleodii* strain isolated from copper coupons and three laboratory mutants grown long-term at elevated copper levels.

## GENOME ANNOUNCEMENT

A marine bacterium was isolated from copper coupons at the Naval Research Laboratory (Key West [KW], FL) seawater corrosion test facility which demonstrated the ability to generate copper nanoparticles. The isolate was initially maintained in artificial seawater medium supplemented with elevated concentrations of copper (1 to 2.75 mM). Preliminary phylogenetic analysis based on sequencing of the 16S rRNA and gyrase subunit B genes identified it as a strain of *Alteromonas macleodii*, a ubiquitous marine gammaproteobacterium. The species clusters by molecular and phenotypic analyses into two ecotypes: one from surface waters and the second from deep water, primarily in the Mediterranean (1). The KW isolate clusters within the surface ecotype. Previous comparative genomic analysis of three surface isolates and a deep-sea isolate revealed that while synteny was well-preserved among the strains, all also possessed multiple genomic islands (GIs) with differential gene content, including several metagenomic islands (2). All surface isolates contained at least one GI specific for metal resistance. *Alteromonas* spp. have previously been shown to be some of the early colonizers of copper-based antifouling paint (3). Due to the prevalent use of copper in the marine environment, primarily as an algaecide (4) in water treatment and as an antifouling agent on ship hulls (5), the adaptive capabilities of the isolate over time to elevated copper levels were examined here at the genomic level.

Subcultures were established from the initial isolate in which copper concentrations were increased in 0.25 mM increments through multiple generations. In addition to the original isolate (CUKW), whole-genome sequencing was performed on three of the subcultures following six months of repeated transfers: two grown continuously with 3 mM copper (KCC01 and KCP01), and the third with 4 mM copper (KCPu01). Genomic DNA was extracted using the DNeasy blood and tissue kit (Qiagen, Valencia, CA), according to the manufacturer’s instructions for Gram-negative bacteria, and included the optional RNase treatment. The initial proteinase K incubation was performed for 2.5 h. The DNA was evaluated using a Qubit double-stranded DNA broad-range reagent kit (Life Technologies, Foster City, CA) with a Qubit fluorometer (Thermo Fisher Scientific, Waltham, MA) and the NanoDrop-2000 (Thermo Fisher Scientific). Whole-genome sequencing of the four strains was performed by the University of Maryland, Institute for Genome Sciences (IGS) using the Illumina MiSeq platform, generating 300-bp paired-end reads. *De novo* assembly was conducted using SPAdes version 3.6 (6). The IGS Annotation Engine was used for structural and functional annotation of the four draft genomes (http://ae.igs.umaryland.edu/cgi/index.cgi) (7). The Manatee tool was used for viewing the data (http://manatee.sourceforge.net). *A. macleodii* strain ATCC 27126 (accession no. CP003841.1), representative of the shallow-water ecotype, served as the reference genome. Characteristics of the assemblies and annotations are described in Table 1. Variant analyses among these strains and *A. macleodii* strains representative of shallow and deep ecotypes as well as comparative genomic analysis between the original isolate and KCC01 will be described in a future publication.

**TABLE 1:** Summary characteristics of whole-genome assembly and annotation

Strain	Mean coverage (×)	No. of contigs	Genome size (bp)	*N*_50_ (bp)	G+C content (%)	No. of transcripts + RNA	Accession no.
CUKW	102	78	5,250,689	498,754	44.6	4,991	MIPW00000000
KCC01	180	74	5,244,637	498,754	44.6	4,982	MIPX00000000
KCP01	134	86	5,249,355	499,040	44.6	4,999	MIPY00000000
KCPu01	172	70	5,249,355	498,754	44.6	4,987	MIPZ00000000

### Accession number(s).

The whole-genome shotgun projects have been deposited in the DDBJ/ENA/GenBank under the accession numbers listed in Table 1. The versions described in this paper are their first versions.
